# Evaluation of the Cherokee Nation Hepatitis C Virus Elimination Program — Cherokee Nation, Oklahoma, 2015–2020

**DOI:** 10.15585/mmwr.mm7222a2

**Published:** 2023-06-02

**Authors:** Whitney Essex, Molly Feder, Jorge Mera

**Affiliations:** ^1^Infectious Diseases, Cherokee Nation Health Services, Tahlequah, Oklahoma; ^2^Cardea Services, Seattle, Washington.

Approximately 2.4 million persons in the United States have hepatitis C virus (HCV) infection, and 66,700 acute HCV infection cases were estimated for 2020 (*1*,*2*). American Indian or Alaska Native (AI/AN) persons are disproportionately affected by HCV infection and experienced the highest rates of acute HCV infection (2.1 cases per 100,000 persons) and HCV-associated mortality (10.17 per 100,000 persons) in the United States during 2020 (*1*). During 2015, Cherokee Nation Health Services (CNHS) in Oklahoma implemented an HCV elimination program, which includes universal HCV screening, primary HCV workforce expansion, and harm reduction services (*3*). To assess progress 5 years after program initiation, CNHS analyzed deidentified health record data. During November 1, 2015–October 31, 2020, a total of 1,423 persons received a diagnosis of HCV infection. Among these persons, 1,227 (86.2%) were linked to HCV care, and 871 (61.2%) initiated HCV treatment; 702 (49.3%) returned for their 12-week post treatment completion visit, at which time 698 (49.1%) had achieved laboratory-confirmed sustained virologic response (SVR), defined as undetectable HCV RNA at ≥12 weeks after completion of treatment (SVR12). Although CNHS has linked the majority of persons diagnosed with HCV infection to care, and those who returned for the SVR12 visit had high cure rates (99.4%), treatment initiation was lower than expected. Future activities should prioritize addressing gaps in treatment initiation after linkage to care and confirmation of hepatitis C cure with SVR12 testing.

Cherokee Nation is the largest AI/AN nation in the United States, spanning 14 counties in Oklahoma and including more than 450,000 registered Cherokee citizens (*4*). CNHS is the largest tribally operated health system in the United States, providing health care for over 100,000 AI/AN persons in 11 health care facilities across the reservation (*5*). During 2015, CNHS initiated an HCV elimination program to improve HCV screening, treatment, and cure. CNHS has published cascades of care, which document the progression of persons through the stages of HCV care, from diagnosis to treatment and cure, beginning before and continuing through 22 months after the start of their program (*3*,*6*,*7*). This report describes the most comprehensive CNHS HCV cascade of care, including five years of program data.

To assess progress of the HCV elimination program, CNHS extracted and analyzed deidentified data collected through the CNHS electronic health record system and HCV treatment database. The reported cascade of care is based on a modified version of the Consensus HCV Cascade of Care* (*7*); treatment completion was not included in the cascade as a distinct stage of care, but completion of HCV treatment among those included in the cascade was assessed. Persons included in this analysis had HCV RNA detected during November 1, 2015–October 31, 2020, and were thought to be alive as of October 31, 2020. Records of persons who received a diagnosis of HCV infection before November 1, 2015, and who had not received treatment as of this date, were also included. SVR12 visit and results were included in the cascade of care when those outcomes occurred by April 30, 2021.

Diagnosis of HCV infection was defined as receipt of a detectable HCV RNA test result. Linkage to HCV care was defined as undergoing an evaluation by a CNHS HCV-trained provider. HCV treatment initiation was defined as documentation 1) by the provider that treatment commenced, or 2) that the prescription was picked up from the pharmacy. The SVR12 visit was defined as documentation that a visit occurred to obtain an HCV RNA result within the study period and ≥12 weeks after the end of treatment. Achieving laboratory-confirmed SVR12 was defined as receipt of an undetectable HCV RNA test result at ≥12 weeks after completion of treatment. Although not included in the cascade of care, treatment completion, defined as documentation by the provider that treatment was completed, or that all prescription refills were picked up from the pharmacy, was assessed. Persons who completed treatment and were assessed for an HCV RNA test result after treatment completion but before the SVR12 due date were not included in the last two stages of the cascade of care (i.e., an SVR12 visit and laboratory-confirmed SVR12).

Sex, age, and presence of advanced liver disease (ascertained using noninvasive liver staging methods, as identified by serologic biomarkers [fibrosis-4 index >3.25^†^]) were also assessed. Treatments during this period consisted of interferon-free, all oral, direct-acting antivirals).

Progress along each step of the cascade of care was assessed by calculating 1) the proportion of persons who completed each step among the population of persons with diagnosed HCV infection, and 2) the proportion of persons at each step who moved to the next step. IBM SPSS Statistics (version 19; IBM Corp.) was used to conduct all analyses. Because this activity was considered a surveillance and public services delivery program, and the data were collected in the context of clinical care,^§^ it was deemed exempt from review by the Cherokee Nation Institutional Review Board. This activity was reviewed by CDC and was conducted consistent with applicable federal law and CDC policy.^¶^

Among 1,423 persons who received a diagnosis of HCV infection during November 1, 2015–October 31, 2020, and who had available demographic data, 870 (61.1%) were male, and 545 (38.3%) were female; 351 (24.7%) were aged 31–40 and 370 (26.0%) were aged 51–60 years. A total of 189 (13.3%) persons met criteria for advanced liver disease or cirrhosis (Table).

**TABLE:** Characteristics of persons with hepatitis C virus infection (N = 1,423) and treatment and outcome indicators — Cherokee Nation Health Services Hepatitis C Virus Elimination Program, Oklahoma, November 2015–October 2020

Characteristic	No. (%)
**Overall**	**1,423 (100.0)**
**Sex**	
Female	545 (38.3)
Male	870 (61.1)
Unknown	8 (0.6)
**Age group, yrs**	
≤20	16 (1.1)
21–30	236 (16.6)
31–40	351 (24.7)
41–50	312 (21.9)
51–60	370 (26.0)
61–70	118 (8.3)
71–80	13 (0.9)
Unknown	7 (0.5)
**Met criteria for advanced liver disease or cirrhosis (fibrosis-4 index >3.25)**	**189 (13.3)**
**HCV cascade of care outcomes**	
Diagnosis of HCV infection	1,423 (100.0)
Linked to HCV care	1,227 (86.2)
Initiated DAA treatment*	871 (61.2)
Completed DAA treatment	800 (56.2)
Returned for SVR12 visit^†,§^	702 (49.3)
Achieved SVR12 (HCV RNA not detected)	698 (49.1)
Did not achieve SVR12 (HCV RNA detected)^¶^	4 (0.2)

**Abbreviations:** DAA = direct-acting antiviral; HCV = hepatitis C virus; SVR12 = sustained virologic response >12 weeks after HCV treatment completion.

* Does not include 17 persons with HCV infection diagnosed within the study period who initiated and completed treatment after October 31, 2020. Among the 98 persons who completed treatment and did not return for the SVR12 visit, 40 had an undetectable HCV RNA result at treatment completion, and 22 had an undetectable result at their 4-week visit.

^†^ Includes six persons who did not complete treatment but who had a 12-week posttreatment visit and had an undetectable HCV RNA result within the study period.

^§^ Among the 98 persons who did not return for a 12-week posttreatment result, an additional 11 had an undetectable HCV RNA result after the study period.

^¶^ Among the four persons who returned for a 12-week posttreatment visit but did not have an undetectable HCV RNA result, three likely had treatment failure, and one likely had HCV reinfection.

Among the 1,423 persons with a diagnosis of HCV infection, 1,227 (86.2%) were linked to HCV care, and 871 (71.0%) of those initiated HCV treatment. Among persons who initiated treatment, 702 (80.6%) returned for their SVR12 visit, among whom 698 (99.4%) achieved laboratory-confirmed SVR12 (Figure).

**FIGURE:**
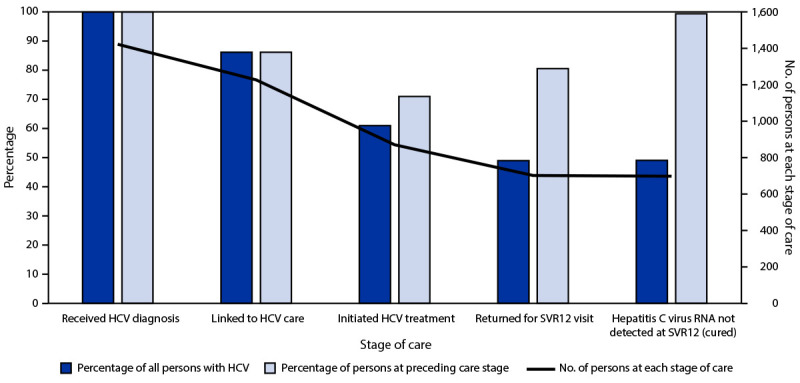
Cascade of care among persons with hepatitis C virus infection (N = 1,423) — Cherokee Nation Health Services, Oklahoma, November 2015–October 2020 **Abbreviations: **HCV = hepatitis C virus; SVR12 = sustained virologic response >12 weeks after treatment completion.

Among the 871 persons who initiated treatment, 800 (91.8%) completed treatment. In addition to the 871 persons who initiated treatment during the study period, another 17 persons initiated and completed HCV treatment after the study period concluded on October 31, 2020; to align with consensus definitions (*7*), these 17 persons were included in the diagnosis and linkage to care levels but excluded from subsequent cascade levels. Among 98 persons who completed treatment but did not return for their SVR12 visit, 40 (40.8%) had evidence of SVR before a 12-week posttreatment visit was due. These results were also not included in the cascade of care.** Eleven of the 98 persons who did not return for an SVR12 visit during the study period had undetectable HCV RNA test results outside of the study period. To align with consensus definitions (*7*), these 11 persons were included in all cascade levels, except the SVR12 visit (Table).

## Discussion

These findings align with those published in a 2022 global systematic review of HCV elimination activities (*8*). Five years after implementing the CNHS HCV Elimination Program, approximately 86% of persons who received a diagnosis of HCV infection were linked to care; however, only 61% initiated treatment, 56% completed treatment, and just under 50% achieved SVR12. Among those who initiated treatment and returned for SVR12 visits, 99.4% were cured. Given the high rate of treatment success with direct-acting antivirals, it is likely that the majority of persons who initiated treatment were also cured (*9*). Thus, although linkage to care has been successful, treatment initiation continues to be a barrier to achieving HCV elimination within the Cherokee Nation.

There are several potential explanations for the gap from HCV treatment evaluation to treatment initiation within the CNHS program. First, Oklahoma Medicaid did not cover hepatitis C treatment for persons with fibrosis scores of F0 or F1 (little to no scarring) until 2018 (*10*). In addition, for all payor types, a previous authorization was required, and although HCV evaluation occurred, several weeks to months might have lapsed before HCV treatment medication became available (*10*). Further, some payors required evaluation by a specialist or that the prescription be written in consultation with a specialist, further delaying treatment initiation (*10*). These delays might have led to some persons falling out of care.

The findings in this report are subject to at least four limitations. First, because this evaluation was conducted among persons served by one Tribal health system, findings might not be generalizable to persons served by other health systems. Second, this evaluation relied on consensus cascade definitions (*7*) that differed from CNHS’s previously published cascades of care and, as a result, these findings are not directly comparable. Third, persons included in this study might have received care outside of CNHS, leading to underreporting of true cascade outcomes. Finally, the COVID-19 pandemic overlapped with the final seven months of this evaluation. Although it is impossible to fully ascertain the effects of COVID-19 on the results of this evaluation, the pandemic might have reduced the numbers of persons attending care visits, initiating treatment, and obtaining laboratory tests to monitor viral load, including SVR12. Despite these limitations, these findings are important because of the disproportionate impact of HCV infection and lack of HCV research among AI/AN persons.

To achieve HCV elimination, the reasons for the gaps at each stage of the cascade of care need to be addressed, especially the delay in the acquisition of hepatitis C medications. For CNHS, emphasis on treatment initiation should be a priority. Future research should explore barriers to linkage to care, initiating treatment after HCV evaluation, completing treatment, and returning for the SVR12 visit among AI/AN persons, as well as interventions to address these barriers.

**Summary**

**What is already known about this topic?**

American Indian and Alaska Native (AI/AN) persons are disproportionately affected by hepatitis C virus (HCV) infection.

**What is added by this report?**

Five years after implementing a hepatitis C elimination program, Cherokee Nation Health Services (CNHS) had diagnosed hepatitis C in 1,423 persons, 86% of whom were linked to care. Although only 61% initiated treatment, 99% of those who completed treatment were cured. Barriers to HCV treatment initiation include lack of access to direct-acting antivirals at the time of HCV evaluation.

**What are the implications for public health practice?**

CNHS’s Hepatitis C Elimination Program can be used as a model for other health systems serving AI/AN persons; however, barriers to HCV treatment initiation need to be addressed to achieve HCV elimination.

## Footnotes

* The consensus cascade of care definitions recommend that reporting includes the number and percentage of persons 1) ever infected with HCV (reactive HCV antibody test), 2) diagnosed with chronic HCV infection, 3) started on treatment, and 4) achieving SVR12.

†
https://www.hcvguidelines.org/treatment-naive/simplified-treatment

§
https://www.hhs.gov/ohrp/coded-private-information-or-biospecimens-used-research.html

¶ 45 C.F.R. part 46, 21 C.F.R. part 56; 42 U.S.C. Sect. 241(d); 5 U.S.C. Sect. 552a; 44 U.S.C. Sect. 3501 et seq.

** These persons were excluded because of the possibility that they might have relapsed 12 weeks after treatment discontinuation, when the SVR12 should have occurred. Because they were tested before that date, it is likely they are cured; however, they do not strictly meet the SVR12 definition.

## References

[1] CDC. Viral hepatitis: 2020 viral hepatitis surveillance report—United States, 2020. Atlanta, GA: US Department of Health and Human Services, CDC; 2022. https://www.cdc.gov/hepatitis/statistics/2020surveillance/index.htm

[2] Office of Infectious Disease and HIV/AIDS Policy. Viral hepatitis in the United States: data and trends. Washington, DC: US Department of Health and Human Services, CDC; 2016. https://www.hhs.gov/hepatitis/learn-about-viral-hepatitis/data-and-trends/index.html#:~:text=2.4

[3] Mera J, Williams MB, Essex W, Evaluation of the Cherokee Nation hepatitis C virus elimination program in the first 22 months of implementation. JAMA Netw Open 2020;3:e2030427. 10.1001/jamanetworkopen.2020.30427 33337496 PMC7749444

[4] Cherokee Nation. Osiyo! Tahlequah, OK: Cherokee Nation; 2023. https://www.cherokee.org

[5] Cherokee Nation. Health services: health center and hospital locations. Tahlequah, OK: Cherokee Nation; 2023. https://health.cherokee.org/health-center-and-hospital-locations

[6] Mera J, Vellozzi C, Hariri S, Identification and clinical management of persons with chronic hepatitis C virus infection—Cherokee Nation, 2012–2015. MMWR Morb Mortal Wkly Rep 2016;65:461–6. 10.15585/mmwr.mm6518a2 27172175

[7] Safreed-Harmon K, Blach S, Aleman S, The consensus hepatitis C cascade of care: standardized reporting to monitor progress toward elimination. Clin Infect Dis 2019;69:2218–27. 10.1093/cid/ciz714 31352481

[8] Lazarus JV, Picchio CA, Byrne CJ, A global systematic review of hepatitis C elimination efforts through micro-elimination. Semin Liver Dis 2022;42:159–72. 10.1055/a-1777-6112 35189667

[9] Office of Infectious Disease and HIV/AIDS Policy. Learn about viral hepatitis: hepatitis C basic information. Washington, DC: US Department of Health and Human Services, CDC; 2020. https://www.hhs.gov/hepatitis/learn-about-viral-hepatitis/hepatitis-c-basics/index.html

[10] Oklahoma Health Care Authority. Hepatic disorders 2017 archives. Oklahoma City, OK: State of Oklahoma; 2022. https://oklahoma.gov/ohca/providers/types/pharmacy/prior-authorization/prior-authorization-2017/hepatic-disorders-2017-archives.html

